# A synthetic dataset of different chart types for advancements in chart identification and visualization

**DOI:** 10.1016/j.dib.2024.110233

**Published:** 2024-02-21

**Authors:** Filip Bajić, Marija Habijan, Krešimir Nenadić

**Affiliations:** a University Computing Centre, University of Zagreb, 10000 Zagreb, Croatia; b Faculty of Electrical Engineering, Computer Science and Information Technology Osijek, 31000 Osijek, Croatia

**Keywords:** Chart classification, Chart recognition, Chart analysis, Document analysis, Text recognition and classification, Graphics recognition

## Abstract

We introduce a meticulously curated synthetic chart dataset designed to propel algorithm advancements in data visualization and interpretation. The dataset, tailored for training and testing purposes, encompasses a diverse array of chart types, including but not limited to Area, Bar, Box, Donut, Line, Pie, and Scatter. The data collection process involves a fully automatic low-level algorithm focused on extraction of graphical elements. The algorithm ensures efficiency by restricting input images from featuring three-dimensional representations, incorporating any 3D effects, or including multiple charts in a single image. The dataset is categorized into training and testing subsets, further subdivided based on resolutions and specific chart types. The reuse potential of this dataset is substantial. It serves as a valuable resource for driving algorithmic advancements in data visualization classification and interpretation. Researchers can leverage this dataset for training and testing deep models, enhancing the adaptability of their algorithms. Moreover, it establishes a benchmark for evaluating system performance in handling diverse chart visualizations, allowing for direct comparisons, and fostering advancements in data understanding algorithms. The versatility of the dataset, encapsulating various chart types and resolutions, provides a standardized platform for assessing and comparing the effectiveness of different systems in understanding and decomposing visualizations [1,2,3].

Specifications Table

**Table:** 

Subject	Artificial Intelligence, Computer Science, Data Engineering and Data Science
Specific subject area	Chart images
Data format	Raw
Type of data	Images (.jpg files)
Data collection	The synthetic chart dataset was generated using Python programming language and Matplotlib library, with no reliance on specific instruments. The data collection process employed custom Python scripts to simulate and visualize the dataset. Since the dataset is artificially generated, there were no explicit inclusion or exclusion criteria applied during its creation. Furthermore, normalization procedures were not deemed necessary, given that the values were directly generated through the Python and Matplotlib framework, ensuring a controlled and consistent representation.
Data source location	University Computing Centre, University of Zagreb, 10,000 Zagreb, Croatia
Data accessibility	Repository name: ChartDataset2023: Introducing a Synthetic Dataset Featuring Various Chart Types for Chart Identification and Visualization Data identification number: 10.17632/yf2wz2n8nx.1 Direct URL to data: https://data.mendeley.com/datasets/yf2wz2n8nx/1
Related research article	

## 1 Value of the Data

• The dataset is valuable for driving advancements in algorithms related to data visualization classification and interpretation, providing a diverse and realistic set of charts that challenge systems to handle varied design complexities.
• The synthetic chart dataset serves as a resource for training and testing systems using deep models, enabling researchers to enhance the robustness and adaptability of their algorithms.
• Researchers can reuse the dataset as a benchmark for testing the performance of their own systems in handling diverse chart visualizations, allowing for direct comparisons and advancements in data understanding algorithms.
• By constructing charts from real-world data sources using the Matplotlib library [4], the dataset captures nuances in data visualization, including variations in chart components like titles, legends, and styles, emulating real-world scenarios more accurately.
• The dataset encompasses multiple chart types, providing a diverse range of visualizations making it a versatile tool for evaluating and improving system performance across various chart formats.
• The proposed dataset establishes a common ground for evaluation, offering a standardized platform for assessing and comparing the effectiveness of different systems in understanding and decomposing visualizations.

## 2 Data Description

**Fig. 1:**
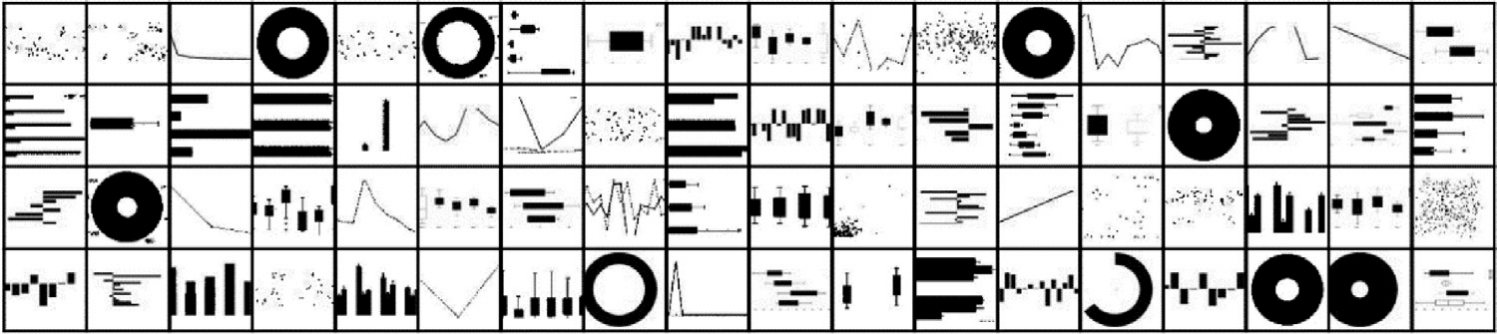
An example of batch from dataset consisting of 72 chart images. The image shows diversity in chart types as well as a diversity in graphical elements used for a specific chart type.

We present a synthetic dataset, meticulously curated for training and testing purposes, encompassing diverse chart types, including Area, Bar, Box, Bubble, Donut, Heatmap, Histogram, Line, Pie, Scatter, Sunburst, Table, and Waffle. Various sub-types are intricately detailed within each chart type, capturing nuances in orientation (horizontal/vertical), stacking, grouping, and specialized characteristics as shown in Fig. 1. The dataset consists of 25,894 images.

The presented dataset serves as a valuable resource for the training, testing, and evaluation of deep learning models, specifically designed for the primary stage of chart image interpretation—namely, chart image classification. The images within the dataset undergo comprehensive processing through a provided algorithm, ensuring a standardized design comprising exclusively graphical elements. This algorithm meticulously eliminates extraneous elements such as colors, numbers, and texts, focusing solely on preserving information pertinent to chart image classification.

In contrast to other datasets prevalent in the research domain, including DocFigure [5], ICDAR [6], ICPR [7], FigureQA [8], PlotQA [9], or SciCap [10], the presented dataset distinguishes itself by its applicability for direct utilization in chart image classification without necessitating additional pre-processing steps. This characteristic significantly reduces the overall system complexity. It is noteworthy that the mentioned datasets are specialized for particular tasks within the realm of chart image interpretation, encompassing functionalities such as question answering systems, chart image summarization systems, and systems involving natural language processing algorithms. These tasks represent more advanced stages in the hierarchy of chart image interpretation. Notably, the presented dataset is crafted to harmonize with these established datasets, providing a cohesive framework for diverse applications within chart image interpretation.

It is noteworthy that, to the best of our knowledge, the presented dataset stands out as a unique contribution in its emphasis on streamlined applicability for chart image classification. This uniqueness positions the dataset as a valuable resource in conjunction with existing datasets, offering researchers a comprehensive toolkit for chart image interpretation tasks.

Organization of dataset in repository [11] is as follows. There are in total 6 folders:

• Training – 1 - 64×64 – subdivided into 8 folders marked as t1, t5, t10, t20, t50, t100, t250, t500. Each subfolder contains 7 folders with *DountPie, HBar, HBox, Line, Scatter, VBar*, and *VBox* .jpg images with a resolution 64×64 pixels.
• Training – 2 - 64×64 – subdivided into folders with *barHoriGrouped, barHoriSimple, barHoriStacked, barVertGrouped, barVertSimple, barVertStacked, boxHoriComplex, boxHoriSimple, boxVertComplex, boxVertSimple, donutComplex, donutSimple, lineComplex, lineSimple, pieComplex, pieSimple, scatterComplex, scatterSimple* .jpg images with resolution 64×64 pixels.
• Training – 105×105 – subdivided into 8 folders marked as t1, t5, t10, t20, t50 t100, t250, t500. Each subfolder contains 18 folders with *DountPie, HBar, HBox, Line, Scatter, VBar*, and *VBox* .jpg images with resolution 105×105 pixels.
• Testing – 64×64 – subdivided into 5 folders marked as set1, set2, set3, set4, set5. Each subfolder contains 18 folders with *barHoriGrouped, barHoriSimple, barHoriStacked, barVertGrouped, barVertSimple, barVertStacked, boxHoriComplex, boxHoriSimple, boxVertComplex, boxVertSimple, donutComplex, donutSimple, lineComplex, lineSimple, pieComplex, pieSimple, scatterComplex, scatterSimple* .jpg images with resolution 64×64 pixels.
• Testing – 105×105 – subdivided into 5 folders marked as set1, set2, set3, set4, set5. Each subfolder contains 18 folders with *barHoriGrouped, barHoriSimple, barHoriStacked, barVertGrouped, barVertSimple, barVertStacked, boxHoriComplex, boxHoriSimple, boxVertComplex, boxVertSimple, donutComplex, donutSimple, lineComplex, lineSimple, pieComplex, pieSimple, scatterComplex, scatterSimple* .jpg images with resolution 105×105 pixels.
• Other – 105×105 – subdivided into 8 folders marked as *Area, Bar, Line, Map, Pie, Radar, Scatter, Venn* with .jpg images with resolution 105×105 pixels.
• Mixed – contains mixed chart images with resolutions 64×64 and 105×105 pixels.

## 3 Experimental Design, Materials and Methods

**Fig. 2:**
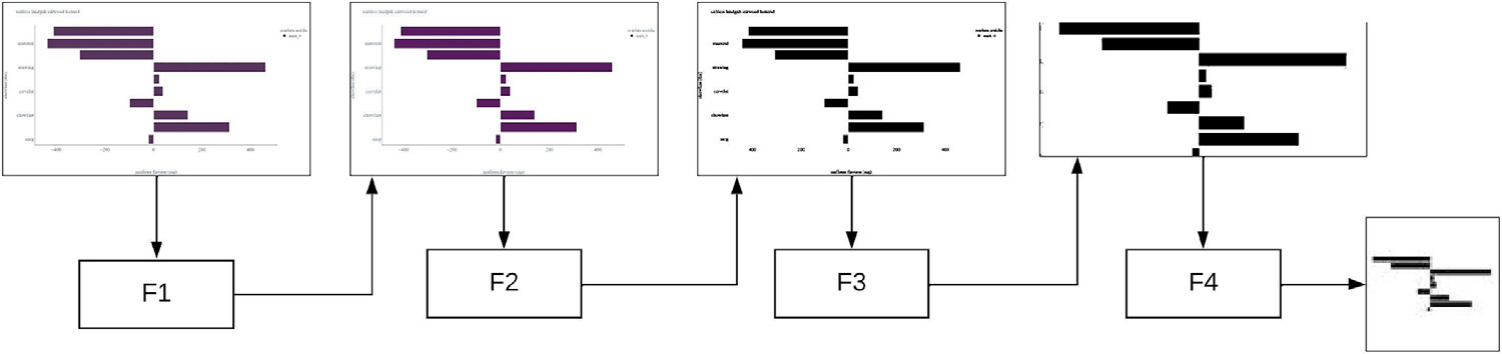
Data acquisition process used for synthetic chart image generation.

A fully automatic low-level algorithm was developed to emphasize shapes of graphical elements on raster images. Firstly, the images should not feature a three-dimensional representation of a chart or incorporate any 3D effects, such as shadows or highlights. Secondly, a single image must not include multiple charts. Lastly, the image should not contain any overlapping elements like watermarks. The acquisition process involves a structured four-step algorithm as shown in Fig. 2.

The first block (F1) is dedicated to image pre-processing, which is a critical phase aimed at preparing and manipulating the input image for subsequent analysis and chart type classification. This step encompasses three filters: color space normalization, gamma correction, and edge enhancement (using a sharpen kernel). Subsequently, the filtered image goes to the second block (F2).

In the second block (F2), the image from the previous block is simplified, that is, the amount of information that the original image contains is significantly reduced. In the second block, three filters are used: gamma-correction, reduction of the number of colors and noise reduction (via median filter). The goal of the second block is to remove colors from the chart image and reduce noise. Color, as a basic building chart element, is not necessary for the classification of the chart and as such does not contain significant information that affects the type of chart. Any type of chart can contain any color, and pie charts usually contain several dozen colors.

The black and white image from the second block is forwarded to the third block (F3). Within the third block, edge elements are removed from the image. Empirical analysis of synthetically created charts, the edge of the chart image most often contains coordinate axes, legend or descriptive labels. The mentioned elements do not contain significant information for chart classification based on graphic elements, and they should be removed. The filter puts focus on the center part of the chart image.

In the last block (F4), the image is prepared for consumption by any system that deals with chart type classification. The output resolution and number of colors are further reduced. The output image retains the original height-to-width ratio, and the empty space is padded with a white background that does not contain significant information. The images are labeled, grouped, and organized within the data interface. The data interface serves as a comprehensive output that applications of interest can readily consume. This methodical approach ensures the systematic acquisition of data from diverse chart images while adhering to rigorous experimental design principles.

The provided algorithm stands out as a unique solution tailored for chart image classification. Its distinctive approach, spanning four sequential processing blocks (F1 to F4), sets it apart in the realm of image pre-processing for subsequent chart type analysis. Notably, this algorithm can be seamlessly integrated with various datasets, enhancing its versatility and applicability.

## Limitations

Despite efforts to simulate real-world scenarios, synthetic datasets may not fully capture the diversity and unpredictability inherent in authentic data. Real-world data often presents unexpected challenges that synthetic datasets might not adequately represent.

## Ethics Statement

The authors state that the present work meets the ethical requirements for publication in Data in Brief. The work does not involve studies with animals and humans.

## CRediT authorship contribution statement

**Filip Bajić:** Investigation, Visualization, Methodology, Data curation, Writing – original draft. **Marija Habijan:** Visualization, Writing – original draft, Writing – review & editing. **Krešimir Nenadić:** Supervision, Writing – review & editing.

## Data Availability

ChartDataset2023: Introducing a Synthetic Dataset Featuring Various Chart Types for Chart Identification and Visualization (Original data) (Mendeley Data).
